# Autophagy-related Proteins in Genome Stability: Autophagy-Dependent and Independent Actions

**DOI:** 10.7150/ijbs.76134

**Published:** 2022-08-21

**Authors:** Ye Zhang, Ran Guo, Shan-Shan Wang, Xiao-You Jiang, Hong-Yan Cui, Yang Guo, Xiao-Yu Song, Qi-Qiang Guo, Liu Cao

**Affiliations:** 1 College of Basic Medical Science, Health Sciences Institute, Key Laboratory of Medical Cell Biology, Ministry of Education, China Medical University, Shenyang, Liaoning Province, P. R. China.; 2 Department of Orthopedics, Shengjing Hospital of China Medical University, Shenyang, Liaoning Province, P.R. China.

**Keywords:** Autophagy-related proteins, Genome Stability, Autophagy, Non-autophagic functions

## Abstract

It is emerging that autophagy-related proteins regulate the adaptive response to DNA damage in maintaining genome stability at multiple pathways. Here, we discuss recent insights into how autophagy-related proteins participate in DNA damage repair processes, influence chromosomal instability, and regulate the cell cycle through autophagy-dependent and independent actions. There is increasing awareness of the importance of these pathways mediated by autophagy-related proteins to DNA damage response (DDR), and disturbances in these regulatory connections may be linked to genomic instability participated in various human diseases, such as cancer and aging.

## Introduction

Actively maintaining the integrity of the genome is essential for the healthy survival of an organism. DNA is the most basic unit of human genetics, and its damage and mutation are usually affected by many extrinsic factors (such as ionizing radiation, ultraviolet radiation, and chemical compounds) and intrinsic factors (such as free radicals produced as by-products of the organism's metabolism and replication errors). Therefore, cells need to evolve efficient mechanisms to sense and repair this damage to ensure their survival.

Autophagy is an essential biological behavior that plays a significant role in recycling cellular components and damaged organelles in response to various stressful conditions, such as metabolic stress and genomic instability. Although autophagy occurs in the cytoplasm, the deletion of autophagy related proteins can also lead to cellular DNA damage and the accumulation of genomic instability. Autophagy-related proteins are significant autophagy executors. Accumulating evidence suggests that autophagy-related proteins can not only regulate various stages of the autophagy pathway, but also participate in cellular biological behaviors in an autophagy-independent manner. Herein, we discuss recent insights into how autophagy-related proteins maintain genome stability through participation in DNA damage repair processes, influencing chromosomal instability, and regulating the cell cycle in an autophagy-dependent and independent manner.

## Autophagy and Autophagy-related Proteins

Organisms are in a dynamic homeostasis of constant renewal. Autophagy is the primary scavenging mechanism in cells by which cytoplasmic components are delivered to lysosomes, where they are degraded. However, the function of autophagy is not just to remove substances from cells; it is a system to maintain homeostasis, help cells complete renewal, and provide energy 1.

**Figure 1:**
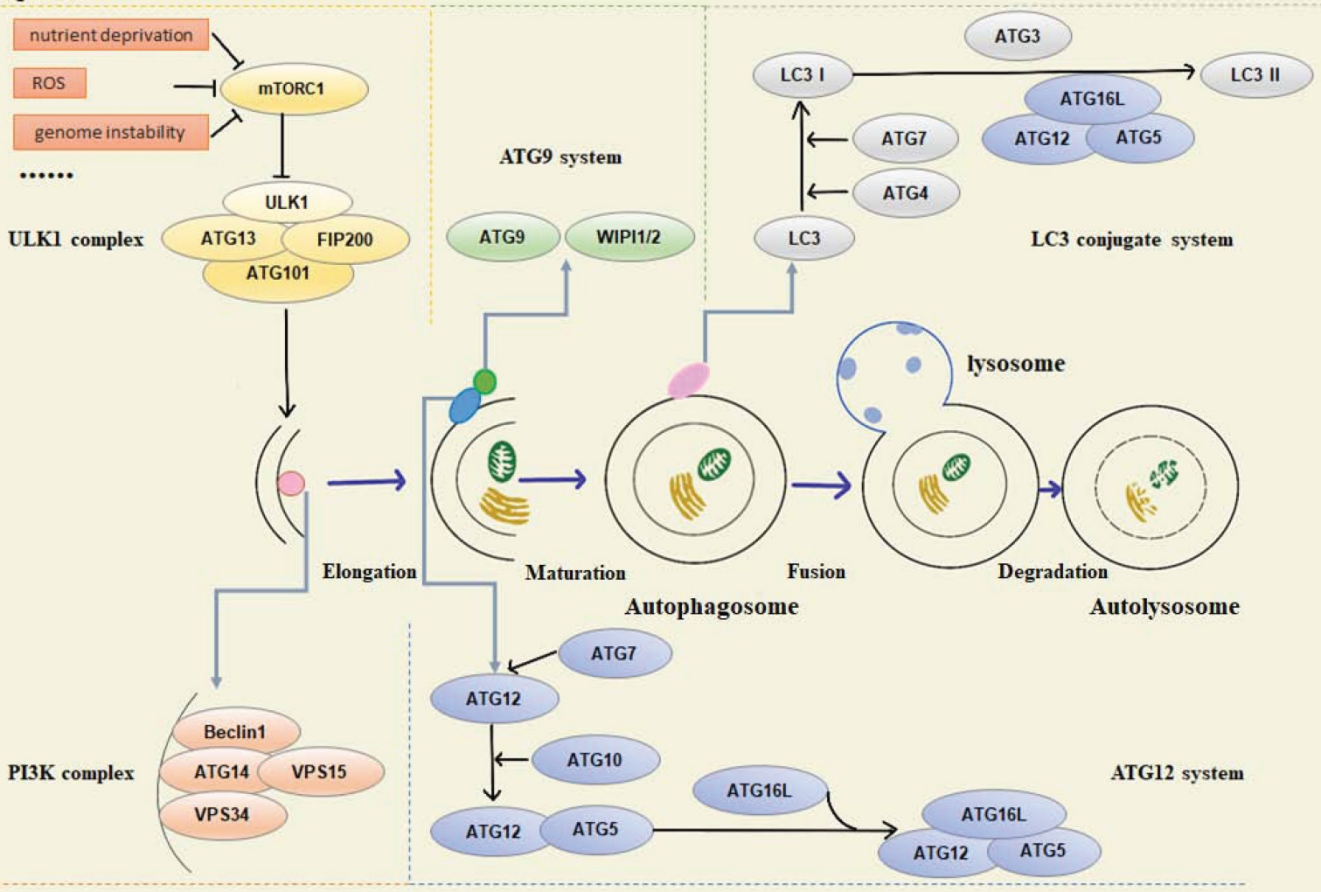
Schematic overview of autophagy-related proteins involved in regulation of the autophagic pathway.

Functionally, various autophagy-related proteins regulate different phases of the autophagy pathway, including origin of autophagy, formation of pre-autophagy structures (PAS), the extension of the membrane and maturity of autophagosomes, transport, recognition of autophagosomes, fusion with lysosomal membrane, and final degradation of autophagic lysosomal contents. In mammalian cells, autophagy is regulated by about 20 core ATG proteins and activated by the mTOR signaling pathway. The proteins involved in the process can be summarized in the following five units: (1) ULK1 kinase complex, including ULK1/2, ATG13, RB1CC1/FIP200, and ATG101, mainly responsible for initiating autophagy, receiving abnormal cell signals, recruiting ATG protein into PAS, and controlling the formation of autophagosomes 2. Signals of intracellular energy depletion activate AMPK to inhibit mTORC1 and initiate autophagy to provide cells with energy and nutrients. This is the initial step of autophagy 3. (2) Autophagy-specific class III phosphatidylinositol 3-kinase (PI3K) complex, including VPS34, VPS15, Beclin1, and ATG14L. Beclin1 interacts with VPS34, which activates VPS34 kinase activity to regulate autophagosome size and quantity 4, 5. (3) ATG9A transportation system, including ATG9A, WIPI1/2, and ATG2A. ATG9A/Atg9 can be phosphorylated by ULK/Atg1, and the recruitment of LC3/Atg8 and WIPI1/2/Atg18 requires phosphorylated ATG9A/Atg9 6. (4) ATG12 ubiquitin coupling system, including ATG12, ATG7, ATG10, ATG5, and ATG16L1. ATG7 activates ATG12, and ATG12 is conjugated to ATG5 via Atg10. The conjugate is then able to be stabilized by the ATG16L protein and form the ATG12-ATG5-ATG16L complex, which is essential for the formation of the LC3-conjugated system 7-9. (5) LC3 conjugate system, including LC3A/B/C, ATG7, ATG3, and ATG4A/B/C/D, is located downstream of the ATG12 ubiquitin coupling system. LC3 localizes to autophagosome membranes following post-translational modifications and is a classical autophagy marker in mammalian cells 10. Both ubiquitin-like systems have a hand in subsequent autophagy processes (Figure 1).

## Genome stability

DNA, the basic unit of human genetics, and its damage and mutation are usually sensitive to environmental factors, including physical factors such as ionizing radiation, ultraviolet radiation, and chemical substances. Meanwhile, there are also many potential dangers in DNA replication and DNA damage repair (DDR); when something goes wrong, unfavorable mutations are likely to occur within the cells. To maintain the integrality and stabilization of the genome, DNA must be protected from damage caused by various environmental factors or spontaneous damage in the process of DNA metabolism. In response to these damages, eukaryotes have evolved the DDR pathway. DDR is a complicated signal transduction pathway that can quickly and accurately sense DNA damage and deliver these signals to cells to activate the response of cells to DNA damage. DDR involves a large number of proteins, and eukaryotic cells have evolved precise strategies to recruit and activate the correct factors at the right place and time 11.

In addition, cells are often exposed to certain stimulatory conditions that impede DNA replication, cause replication fork suspension, and increase fork instability. The resulting DNA replication stress prevents cells from completing DNA synthesis in a timely manner during interphase, leading to chromosomal breakage and genomic instability in mitosis 12, 13. Regulating the operation of replication forks during replication errors is also an essential part of maintaining genome stability.

Cell cycle arrest is one of the most typical cellular response functions activated after DNA damage. It prevents DNA-damaged cells from entering mitosis and provides time for DDR. Cells use cell cycle checkpoints throughout the interphase to protect genomic integrity prior to mitosis and to block the cell cycle in time when unstable states occur. The cell cycle is mainly regulated by cyclin-dependent kinases (CDKs), which drive the G1/S transition and then regulate the DNA replication process in the S phase, which completes most of the DNA synthesis 14, 15. When the cell cycle reaches S phase and G2 phase, mitotic cyclins are expressed, and CDKs activity continues to increase 16.

Improving chromosome instability has become a vital focus of the mitotic process to protect genomic integrity. Telomeres are susceptible to mitotic pressure and are thought to be the region where DDR is initiated. The maintaining telomere stability is essential to protect genome integrity and mitochondrial integrity 17.

It has been proven that autophagy plays an essential role in maintaining genomic stability 18. When cells are under starvation or stress, abnormal mitochondria can produce high levels of ROS, which can lead to DNA damage. Autophagy contributes to the removal of all irretrievably oxidized biomolecules in cells, and the redox homeostasis maintained by autophagy significantly contributes to maintaining genomic stability. This is a crucial reason why autophagy is included in the system for maintaining genomic homeostasis 19. It has also been shown that NDP52 and P62 regulate retrotransposon insertion in the genome through selective degradation of retrotransposon RNA by autophagy, suggesting that autophagy can buffer genetic variation in the physiological sense by degrading retrotransposon RNA 20. However, another study showed that methyltransferase-like 14 (METTL14) positively regulated genome-wide repair by regulating N6-methyladenosine (m6A) methylation modification and DDB2 translation. Under UVB irradiation, METTL14 was downregulated by NBR1-mediated selective autophagy 21. This suggested that autophagy played different roles in regulating genome homeostasis under different circumstances.

However, reports continue to indicate that autophagic proteins also have many non-autophagic functions, including regulating neuronal growth and axon growth, participating in DNA damage responses, and participating in type I IFN signaling 22-26. The mechanism of autophagy proteins regulating genomic homeostasis in the nucleus has attracted a certain degree of attention. Interestingly, autophagy proteins can maintain genome homeostasis not only through autophagy-dependent pathways, but also through autophagy-independent pathways, exerting their non-autophagic functions in the nucleus. This article reviewed the current research on autophagy-related proteins in maintaining genome stability, to figure out the effects of autophagy-related proteins in the nucleus.

## Autophagy-related proteins maintain genome stability through autophagy-dependent pathways

The autophagy pathway plays a vital role in maintaining all aspects of nuclear genome homeostasis, and autophagy-deficient cells are likely to experience genomic instability. DNA damage, chromosomal instability, and cell cycle arrest are common events 27, 28. Activation of autophagy has been shown to reduce genomic instability 29, 30. Recent studies have indicated that autophagy is directly participated in DDR in yeast and mammalian cells, regulating levels of a critical repair protein, RBBP8/CtIP 31. When autophagy is activated, a variety of autophagy-related proteins can directly or indirectly affect genome homeostasis. For example, in mammals, autophagy regulates the protein level of active RHOA by regulating SQSTM1/p62. When autophagy is interrupted, active RHOA accumulates in the region surrounding the intermediate and leads to cytokinesis failure, multinucleation, and aneuploidy 32. We discussed recent insights into how autophagy-related proteins participate in DNA damage repair processes, influence chromosomal instability, and regulate the cell cycle in an autophagy-dependent manner.

### Autophagy function of autophagy-related proteins and DNA damage

Genome stability is essential throughout the life of a cell. Maintaining genomic homeostasis requires constant counteracting of accumulated DNA damage. Unrepaired DNA damage can cause a series of serious consequences, including cell cycle arrest and aging, apoptosis, cellular dysfunction, and mutation accumulation. Therefore, DNA damage repair is crucial in maintaining genomic homeostasis. DNA damage often occurs in the nucleus and is caused by environmental factors (UV light, ionizing radiation) and internal factors (ROS) 33. In addition, when autophagy is impaired, oxidative stress will also lead to the accumulation of DNA damage. Therefore, maintaining intact autophagy is crucial in preventing DNA damage 34.

The mechanism of autophagy involved in DDR has been extensively studied and discussed. What is clear is that DNA damage can activate autophagy pathways and mitigate the state of genomic instability 33. DNA double-strand break (DSB) is a very common incident in DNA damage. Taking DSB as an example of a representative DNA damage event, DSB can activate ATM, which leads to the LKB1-mediated phosphorylation of AMPKα subunit at Thr172. AMPK then activates TSC2 and ULK1, leading to the inhibition of mTOR, ATG1 activation, and autophagosome formation 3.

There is also evidence that autophagy mediated by Unc-51-like kinase 1 (Ulk1) and LC3 also plays an important role in combating genomic instability. Ulk1 and LC3 are two core components of autophagy. Ulk1 is involved in the induction of autophagy, while the phosphatidylethanolamine-conjugated form of LC3 is currently the most accurate autophagosome marker protein 35. It has been reported that both LC3 and pUlk1 have nuclear localization. When LC3 and Ulk1 are silenced, autophagy is inhibited, and micronuclei, a diagnostic marker of genomic instability, is accumulated; silencing either one leads to an increase in the generation frequency of micronuclei and promote genomic instability 36. Gao et al. found that in response to DNA damage, p53 increased the transcriptional level of Ulk1/Ulk2 and initiated sustained autophagy. By upregulating Ulk1/2, p53 promotes Ulk1/2 and other autophagy-initiating components such as ATG13, FIP200, and ATG101 to produce Ulk1 kinase complex, preparing for the occurrence of autophagy. In this process, AMPK and sesn1/2 levels are also upregulated, resulting in inhibition of mTOR kinase activity, thereby eliminating the inhibition of mTOR on autophagy. Disinhibition of mTOR further increases autophagic flux, resulting in sustained high levels of autophagy. If DNA damage cannot be repaired and cells are in a sub-death state of high-intensity autophagy for a long time, continuous autophagy will lead to non-apoptotic cell death 37.

FIP200 is an adhesion plaque kinase that is involved in autophagy. Studies have shown that FIP200KO MEFs show nuclear γ-H2AX staining after exposure to ionizing radiation (IR), indicating that DDR is defective at this time, and FIP200 is involved in DNA damage repair. Similar results showed that autophagy was inhibited in the presence of other DNA damage inducers such as camptothecin and etoposide, as well as in the use of 3-methyladenine. These suggest that FIP200 is likely to be involved in DDR through the autophagy pathway 38.

Beclin 1 is a key member of the autophagy protein family, and functions as a core component of the class III PI3K/Vps34 complex required for autophagosome formation and maturation 39. UVRAG products can be associated with the Beclin 1/Bcl-2/PI(3)KC3 complex, in which UVRAG and Beclin 1 interact and codependently induce autophagy through their coiled-coil domain (CCD) 40. UVRAG interacts with Beclin 1 to modulate the DNA damage signal, and Beclin 1 siRNA can significantly reduce the expression level of UVRAG. Experiments have shown that UVRAG ΔCCD mutant shows substantially damaged binding to Beclin 1, resulting in a more severe extent of radiation-induced DSBs. In mammalian cells, DSB is mainly repaired by two biological mechanisms, non-homologous end joining (NHEJ) and homologous recombination (HR). The core of HR repair DSB is Rad51, which is to promote homologous pairing between DNA double strands; 53BP1 is a sensor of DNA damage and a promoter of non-homologous end joining (NHEJ). Following irradiation treatment, 53BP1 was significantly enhanced in Beclin 1, UVRAG or ATG5 knockdown cells compared to control cells. This suggests that the involvement of Beclin 1 in DDR may be related to autophagy 41. In addition, when the allele of Beclin1 is lost, autophagy deficiency will activate the DNA damage response in breast tumor cells, promote gene amplification, and cooperate with apoptosis defects to promote the occurrence of breast tumors. The research results also showed that Beclin 1 could play a role in resisting DNA damage or DNA damage repair 27.

Autophagy has been shown to occur in the cytoplasm. How does it participate in the function of the nucleus? Research shows that p62 plays a vital role in cytoplasmic communication during autophagy in regulating nuclear genome homeostasis 42, 43. P62 is amomentous autophagy adaptor protein that binds to ubiquitinated protein polymersand delivers them to autophagosomes. In the cytoplasm, p62 acts as a receptor on autophagosomes to direct autophagy and autophagosome maturation. When p62 is reduced, cellular polyploidy increases, and autophagic maturation is impaired. Mitochondrial coding protein LRPPRC is combined with one of the microtubule-associated protein family MAP1S to promote autophagy initiation and development. Liver-specific deletion of LRPPRC results in a decrease in p62, which causes autophagy damage that negatively affects cells 44. P62 has nuclear localization signals and nuclear export signals that enable it to shuttle between the cytoplasm and nucleus. In the nucleus, the repair process of DNA damage is affected by p62, which is specifically located in the nucleus. The inhibition of autophagy will lead to an increase in the level of nuclear p62, which in turn leads to a decrease in chromatin ubiquitination. This ubiquitination is essential for the recruitment of downstream factors of the DSB repair pathway, such as the 53BP1 and BRCA1/BARD1 complex 45. In terms of mechanism, the ubiquitination reaction induced by DSB is triggered through the RNF8-dependent adjoint of ubiquitin on histone H1 46. RNF8 is recruited to the injury site to initiate H2A/H2AX ubiquitination via K63. Subsequently, RNF8 amplifies this cascade by recruiting an E3 ligase, RNF168, which promotes the monoubiquitination of histone H2A/H2AX to transmit DNA damage signals and activate the DDR program 47. However, the interaction of the LIM-binding (LB) domain of p62 and the MU1 domain of RNF168 inhibits the E3 ligase activity of RNF168, resulting in damaged chromatin ubiquitination and decreased recruitment of DNA repair proteins after DNA damage 48. Furthermore, when autophagy is inhibited or defective, there is another regulatory mechanism through which p62 accumulation affects DNA repair which has been reported in the literature through proteasomal degradation of filamin A (FLNA) and RAD51 in the nucleus to inhibit HR-directed DSB repair. The role of FLNA in DDR is to recruit RAD51 through interaction with BRCA1/2. The interaction of p62 and FLNA promotes proteasomal degradation of FLNA and RAD51 in the nucleus, reduces nuclear RAD51 levels, and slows DNA repair, especially through HR 49. These results suggested that the accumulation of p62 caused by autophagy defects and nuclear import of p62 could disturb DSB repair in both NHEJ and HR pathways. In addition, when autophagy is inhibited, the accumulation of p62 leads to the production of ROS and ER stress. The increase of ROS levels under oxidative stress can also lead to DNA damage. Preventing the accumulation of p62 or ROS can reduce the damage of autophagy-deficient cells 50**.** Clinically, Christoph Burdelski et al. found that this accumulation of p62 caused genomic instability and promoted rapid proliferation of prostate cancer cells, leading to tumor recurrence 51. The phenomenon of p62 accumulation caused by inhibition of autophagy has been confirmed in many cancer cells, such as oral cancer, colon cancer, breast cancer, and so on 52, 53.

Studies have shown that both Ulk1 and LC3 participate in DSB. γ-H2AX (phosphorylated histone H2AX Ser139) is a classic marker of DSB. Both LC3-II and pUlk1 show nuclear localization, and can combine with γ-H2AX, Rad51, or PARP-1 to maintain genomic stability. Sunitinib can increase the level of γ-H2AX and enhance autophagic flux. When Sunitinib is administered, the binding of LC3, pUlk1 and Rad51 is weakened, indicating that autophagy may limit the role of LC3 and pUlk1 in DSB repair 36.

**Figure 2:**
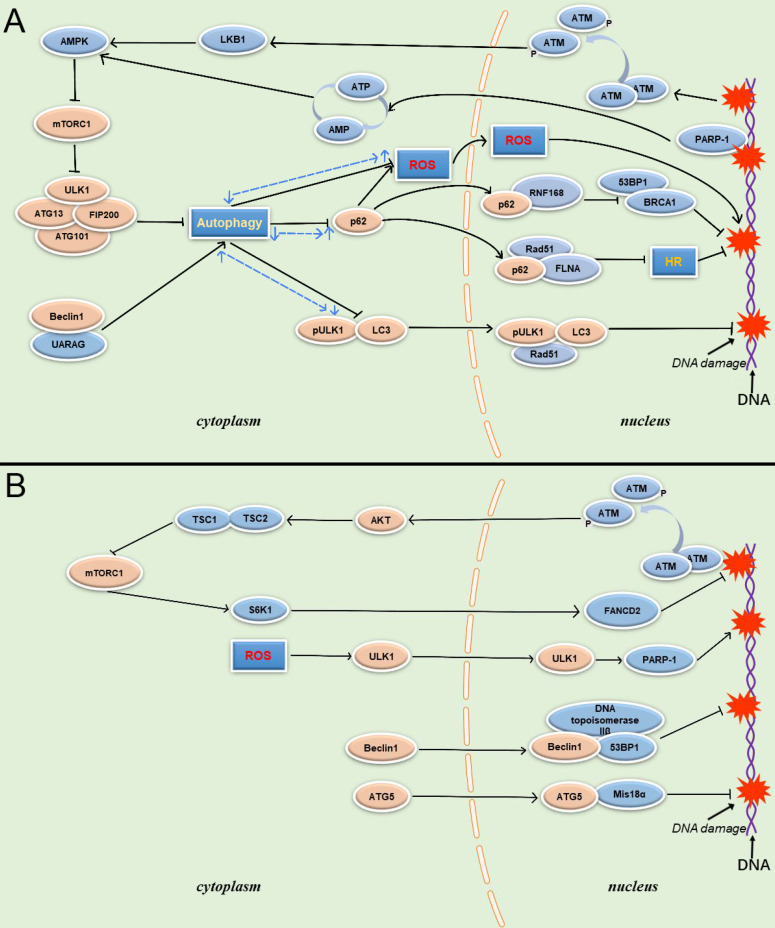
** Schematic overview of autophagy-related proteins involved in DNA damage through the autophagy pathway and non-autophagic pathway. (A)** Autophagy related proteins involved in DNA damage through the autophagy dependent pathway. **(B)** Autophagy related proteins involved in DNA damage through the non- autophagic pathway.

PARP-1 is another protein involved in autophagy regulation during DDR, and plays a pivotal role in the interaction between autophagy and DDR. PARP-1 is an important sensor of DNA breaks, and its catalytic activity is activated up to 500-fold on the site of DNA breaks. It is also an important recruitment molecule, and can concentrate the key molecules required for DNA fracture repair at the damaged site in a short time 54. Meanwhile, overactivation of PARP-1 leads to ATP depletion, and AMPK can activate TSC1/2 to inhibit mTOR after sensing energy consumption to activate autophagy, enabling cells to obtain nutrients and energy. However, in response to DNA damage, the energy collapse caused by the over-activation of PARP-1 and induced high intensity continuous autophagy is a death signal for the cell. Although autophagy is a protective factor in the response to DNA damage, PARP-1deficient cells are better rescued than cells undergoing PARP-1induced persistent autophagy, resulting in an overall reduction in cell death 55 (Figure 2).

### Autophagy function of autophagy-related proteins and chromosome instability

In the nucleus, chromosomal instability is inextricably linked to DNA damage. Chromosome instability (CIN) refers to the abnormal condition of chromosome integrity or chromosome number in the nucleus. CIN cells produce higher levels of ROS, leading to DNA damage and cell death. In CIN cells, autophagy activity also becomes more sensitive. Knockout of autophagy-related proteins Atg1 or Atg18 resulted in a remarkable increase in oxidative stress and DNA damage levels in CIN cells 56, indicating that autophagy plays a rescued role in CIN cells, and the improvement of autophagy level promotes the survival of CIN cells.

Telomeres are essential structures that chromosomes have evolved to maintain their stability. Telomeres are tandem TTAGGG DNA repeats that preserve genomic stability by recruiting protein complexes called shelterins, which protect the ends of chromosomes from being identified as DSBs 57. Due to the loss of telomere sequence caused by replication shortening, the dysfunctional telomeres will activate the typical DNA damage response. Cells with telomere dysfunction have increased chromosomal fusion, resulting in genomic instability, which macroscopically contributes to the pathogenesis and progression of cancer. Autophagy suppresses this chromosomal instability by maintaining cellular metabolic homeostasis and inhibiting tumor progression 28, 58.

Autophagy can limit chromosomal instability, which could also explain its tumor suppressor function from a genetic perspective. Beclin1^+/-^ cells are deficient in autophagy and susceptible to stimulation by metabolic stress. This innate defect in autophagy-deficient cells limits their survival; however, the cells increase the rate of gene mutation and chromosomal instability in order to survive, and even develop aneuploidy, a sign of tumorigenesis 28. Some studies treated Beclin 1 as a dose-dependent tumor suppressor gene and found that Beclin 1^-/-^ mice had early embryonic death. Meanwhile, Beclin 1^-/-^ mutant ES cells had serious defects in autophagy, and Beclin 1^+/-^ mutant mice had high incidence of spontaneous tumors 59. The monoallele deletion of *BECN1* also accelerated tumorigenesis in mice in the ovarian cancer model, suggesting that autophagy is a suppressor of ovarian cancer 60.

**Figure 3:**
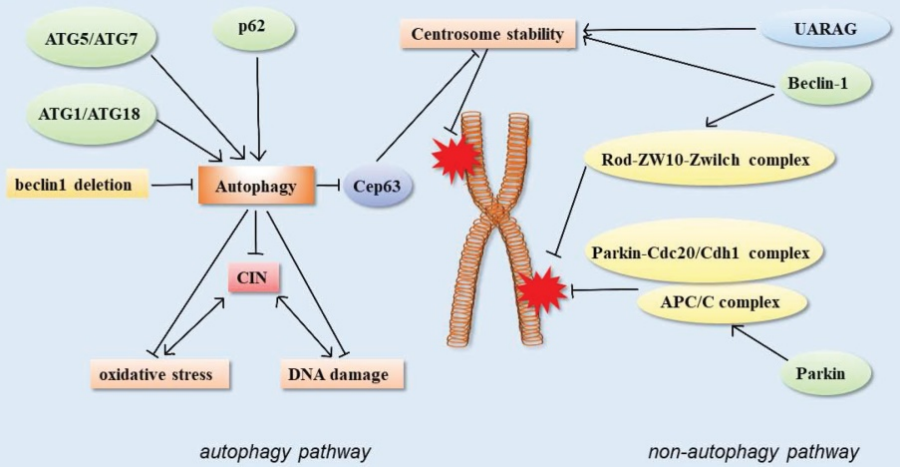
Schematic of autophagy-related proteins involved in regulating chromosome stability through the autophagy pathway and non-autophagic pathway.

In addition, the centrosome number is associated with correct chromosome separation and genomic stability during mitosis. The ubiquitin-proteasome system is considered to be the main way to regulate the number of centrosomes, but recent studies have shown that autophagy also regulates the number of centrosomes. Autophagy-deficient cells contain multiple Cep63 dots, which increase the number of centrosomes. In wild-type cells, Cep63 is eliminated by p62-mediated autophagy. In mouse models, p62^ -/-^ MEFs were also found to carry more centrosomes than normal. *In vivo*, p62^-/-^ mouse hematopoietic cells had autophagy defects, and also contained multiple centrosomes. These results suggested that p62-mediated autophagy could regulate chromosome stability by regulating centrosome number 61 (Figure 3).

### Autophagy function of autophagy-related proteins involved in cell cycle regulation

If DNA damage is not repaired for a while, cells escape from the growth-inhibitory phase, activating cell cycle checkpoints, resulting in a cell cycle arrest state that allows time for DNA damage repair, known as cell cycle arrest 62. After genome integrity is repaired, cells are released from cell cycle arrest. However, if DNA damage cannot be efficiently restored, cells will either continuously stagnate until death, or begin to replicate with unstable genomes. Autophagy-related proteins act on cell cycle regulation through the autophagy pathway, maintain genome stability, and regulate cell proliferation.

In *C.elegans*, bec-1/Beclin1, atg-18/wipi1/2 all promote the cell cycle process. Autophagy mediated by Bec-1/Beclin1 may be effective as an exocrine system. Since autophagy genes are observed from *C. elegans* to human, the function of bec-1/Beclin1 in *C. elegans* is a good reference for the proliferation of human tumor cells 63. Genomic instability can directly or indirectly cause tumorigenesis. In recent years, studies have found that Beclin-1 induced autophagy can inhibit tumorigenesis through various mechanisms. Mechanically, this protective behavior may be related to three pathways. First, Beclin-1 induce autophagy and cell cycle arrest 64-66. Second, as above, Beclin-1 expression places restrictions on chromosomal instability and reduces the occurrence of genetic mutations and DNA damage 28. The third mechanism is related to autophagy-induced immune responses 67. The Autophagy inhibitor 3-MA, acting on the Beclin 1/PIK3C III complex significantly, delays IR-induced G2/M phase arrest when cells are exposed to IR, suggesting that Beclin 1 may be associated with mitosis and the cell cycle 63. A possible mechanism is that IR enhances the combination of Beclin 1/PLK1 and Beclin 1/CDC25C, suggesting that Beclin 1 may be involved in IR-induced G2/M arrest by combining with PLK1 and CDC25C 68.

ULK1-ATG13, the most upstream of the autophagy initiation complex, is phosphorylated by mTORC1 and AMPK to induce autophagy. Moreover, ULK1 and ATG13, the substrates of cdK1-CCNB/Cyclin B, are essential for cell cycle. Cdk1-induced phosphorylation of ULK1-ATG13 promotes autophagy and cell cycle progression. Studies have shown that double knockout of ULK1 and ATG13 significantly inhibits cell cycle and tumor cell proliferation *in vitro* and *in vivo* 69. Ulk1-ATG13 not only provides a molecular mechanism to maintain autophagy, but also link autophagy to cell cycle regulation 70.

An interesting study showed that overexpression of Atg7 enhanced neural crest cell production in unilateral developmental neural tubes of chicken embryos. The Atg7 gene is located upstream of autophagy, which is responsible for inducing autophagy and removing damaged macromolecules and organelles when cell homeostasis is under various pressures 71. In neural tube cells, the upregulation of the Atg7 gene can activate autophagy. At the same time, the upregulation of Atg7 can significantly accelerate the cell entering the S phase, which means that Atg7 regulates cell cycle progression 72. However, the specific mechanisms and roles of Atg7 in cell cycle regulation seem to depend on the situation. Atg7 is a decisive factor in the arrest of the unstable cell cycle in serum-deficient and amino acid-deficient mouse embryonic fibroblasts 73. However, other studies have shown that Atg7 can inhibit the effect of the CDK inhibitor p27 and control cell proliferation 74. Wang et al. believed that, in neural stem cells (NSC), Atg7 and P62 might inhibit the G1 to S phase of the cell cycle through the autophagy pathway, thus inhibiting the differentiation of NSCs and promoting cell survival 75. Meanwhile, the inhibition of autophagy by Atg7 deficiency may accelerate apoptosis and arrest the cell cycle in G0/G1 phase under glucose starvation 76.

The autophagy protein system is extensive. In addition to the above-mentioned vital proteins located upstream, other autophagy-related proteins participate in the regulation of the cell cycle. ATG10 is highly expressed in gastric cancer (GC) and may act as an oncogene to regulate the cell cycle and promote abnormal cell proliferation 77. ATG4 is a kind of cysteine protease that is necessary for the formation of autophagosomes. ATG4B is the mammalian ortholog of yeast ATG4. In various cancer cells, the knockout of ATG4B can inhibit autophagy and induce cell cycle arrest, the main mechanism of which is to trigger the LKB1-AMPK energy response pathway to stop the G1/S period transition 78.

MTORC1 is a pivotal regulator of the G1 phase and autophagy. Regulation of the G1 phase is usually achieved by regulating cyclin D1 mRNA and protein ubiquitin-proteasome degradation. Everolimus specifically inhibits the mTORC1 signaling pathway and activates the autophagy pathway, blocking cell cycle progression at the late stage of G1 phase. There are two mechanisms: first, mTORC1 inhibitors trigger cyclin D1 nuclear output, ubiquitination, and 26S proteasome degradation by activating GSK-3β kinase 79. Second, in addition to the ubiquitin-proteasome pathway, Everolimus can also promote cyclin D1 degradation through the autophagy pathway 80.

**Figure 4:**
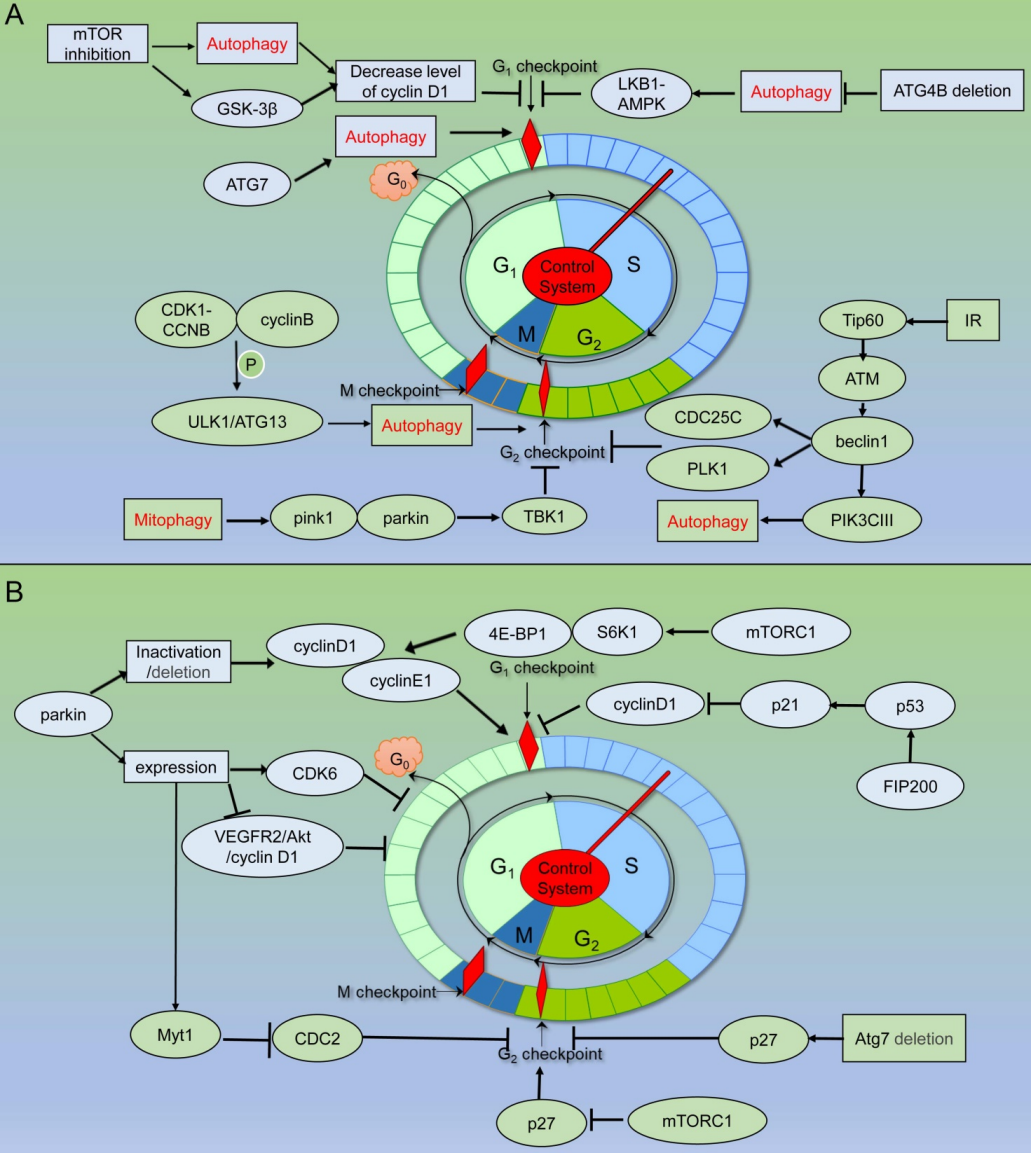
** Schematic of autophagy related proteins involved in cell cycle regulation. (A)** Autophagy related proteins involved in cell cycle regulation through the autophagy dependent pathway. **(B)** Autophagy related proteins involved in cell cycle regulation through the non- autophagic pathway.

PINK1 and Parkin play essential roles as upstream factors in mitophagy targeting the clearance of damaged mitochondria, but their roles in maintaining genome homeostasis have not been explored sufficiently. PINK1/Parkin pathway can activate TBK1, and the phosphorylated TBK1 is isolated from the centrosome to the damaged mitochondria, resulting in G2/M phase arrest. In addition, ATM is an important regulatory protein in DDR. PINK1 and Parkin also interact with ATM genetically, but there seems to be no direct protein communication 81 (Figure 4) (Table 1).

## Autophagy-related proteins maintain genome stability through autophagy-independent pathways

Autophagic proteins are interlinked to form a complete autophagy pathway maintaining cell homeostasis and genome stability. However, more and more studies have offered eloquent proof that the non-autophagy function of autophagy proteins also plays an essential role in cells. These proteins are involved in a wide range of cellular functions 22-26. In the nucleus, autophagy proteins can also participate in teams that control genome homeostasis through non-autophagic pathways. While summarizing that autophagy proteins protect genomic homeostasis through the autophagy pathway, we also determined the mechanisms by which autophagy proteins function through non-autophagy pathways when several of the above-mentioned genomic abnormalities occur in cells.

### Non-autophagic function of autophagy-related proteins involved in DNA damage

Cells complete DNA replication during mitosis, but under the attack of replication inhibitors, DNA replication forks are stalled, genome stability is disrupted, and the mitotic process is interrupted. mTOR is crucial for the resurrection of stagnant replication forks. Protein kinase B (PKB/AKT) has been shown to activate mTORC1 by phosphorylation of the TSC1/TSC2 complex, followed by phosphorylation of S6K and 4E-BP1. Phosphorylation of 4E-BP1 promotes DNA repair and replication fork survival, and the activated S6K1 performs DNA repair through FANCD2 82, 83. ATR-dependent activation of mTORC1 can also regulate nuclear F-actin to promote replication fork repair under replication stress 84. There is an interaction between mTOR and the FA protein pathway, which is the classical repair pathway that regulates replication fork restart. Both can regulate the reboot of stalled replication forks via aphidicolin, an inhibitor of DNA polymerase alpha that causes fork stall and through the same pathway protects newly synthesized DNA strands from degradation by exonuclease 85. However, in another case, the mTOR signaling pathway played a role in accelerating DNA damage caused by deletion of the breast cancer-related gene BRCA1. In BRCA1-deficient cells, mTORC2 is overactivated, so BRCA1-deficient breast cancer cells may be dependent on mTORC2 signaling and more sensitive to its inhibition, further suggesting that mTOR plays a role in driving DNA damage events in tumor cells 86. So how does the mTOR signal mediate DNA damage? Studies have shown that this link also appears to be related to the phosphorylation of AKT. First, AKT can inhibit the activation of mTORC1 by TSC 1/2; at the same time, AKT can dissociate PRAS40, which inhibits mTORC1 autophosphorylation, from mTORC1 components 87, 88. Second, AKT is a direct phosphorylation target of ATM, an important sensor protein in DDR 89. It has been reported that direct DNA damage after rapamycin treatment cannot be detected by pulsed-field electrophoresis, indicating that, when mTOR is inhibited, DDR activation is more likely to occur than significant DNA damage 90.

Promoter hypermethylation leads to the epigenetic inactivation of MLH1 (mutL homolog 1) in the context of CpG island methylation phenotype (CIMP), resulting in DNA mismatch repair deficiency (MMR-D) 91. Autophagy-related gene 5 (ATG5) locates in the cytoplasm and participates in autophagy through interaction with ATG12, promoting the formation of autophagosomes 92. It has been reported that ATG5 in the cytoplasm can translocate to the nucleus. In the nucleus, ectopic ATG5 loses its activity of recruiting ATG12 to participate in autophagy, and exerts a DNA damage function independent of its autophagy activity. After ATG5 enters the nucleus, it interacts with Mis18α and promotes the hypermethylation of the hMLH1 promoter CpG island by promoting the hypermethylation of hMLH1, thereby causing microsatellite instability and DNA mismatch repair deficiency 93.

P62 can also participate in DNA damage through non-autophagy pathways, and the mechanism is associated with high ROS levels. It has been reported liver cells transfected with P62 have increased levels of stem cell marker DLK1, which enhances ROS levels, an important inducer of DNA damage and chromosomal instability 94. P62 induces ROS production by activating NADPH oxidase in a DLK1-dependent manner, thereby compromising genome homeostasis, inducing inflammation, and promoting tumorigenesis 95. Is DNA damage caused by ROS related to autophagy-related proteins? It has been found that after H_2_O_2_ treatment, most of the cytoplasmic ULK1 can localize to the nucleus and regulate the activity of DDR protein PARP1 in a kinase-dependent manner. PARP1 is a core protein in DDR and cell death mechanisms under oxidative stress. By enhancing PARP1 activity, ULK1 promotes the energy consumption and cell death under oxidative stress. This pathway proves that ULK1 can act as a DNA damage factor in the Atg7-independent autophagy pathway, but this view does not deny the possibility that ULK1 promotes autophagy-mediated cell death 96.

Interestingly, some studies have demonstrated that Beclin 1 can be involved in DDR independent of the autophagy pathway 97. Beclin 1 can transfer from cytoplasm to nucleus after cells are exposed to IR. DNA topoisomerase IIβ is one of the most significant proteins interacting with nuclear Beclin 1, and the knockdown of DNA topoisomerase IIβ can inhibit the activity of HR and NHEJ pathways. DNA topoisomerase IIβ and Beclin 1 are able to co-localize with the DSB repair protein p53-binding protein 1 (53bP1) after exposure to IR. It has been speculated that Beclin 1 protects genome stability by cooperating with DNA topoisomerase IIβ. Interestingly, Beclin 1 loses its ability to localize to DNA damage sites when DNA topoisomerase IIβ is completely silenced, suggesting that DNA topoisomerase IIβ might directly recruit Beclin 1 to the DNA damage site, recruit DNA damage repair proteins, and promote DNA damage repair (Figure 2).

### Non-autophagic function of autophagy-related proteins involved in regulating chromosome stability

Monoallelic loss of Beclin 1 has been reported to cause chromosomal disorders, resulting in chromosomal instability 28. However, it remains to be determined whether all of these phenomena are caused by autophagy defects and whether they reflect the non-autophagy-dependent functions of Beclin 1. Increasing evidences suggests that autophagy proteins participate in genome homeostasis regulation through non-autophagy pathways. Studies have demonstrated that Beclin 1 depletion leads to severe chromosomal aggregation defects. The mechanism is related to the decrease in the core components of several kinetochore components, including ZW10, CENP-E, and CENP-F. Among them, Beclin 1 can directly interact with the external kinetochore component Zwint-1. Beclin-1 acts upstream of the Rod-ZW10-Zwilch (RZZ) complex to facilitate the accurate localization of motilin to the spindle during mitosis due to its direct binding to kinetochore constituent proteins 98. In addition, Beclin 1 and/or UVRAG also modulate centrosome stability. Centrosome amplification can lead to spindle malformation and chromosome segregation errors, resulting in chromosomal instability. There was a significant increase in the number of multicentrosomes when Beclin 1 or UVRAG was knocked down, whereas the number of multicentrosomes increased to a lesser extent when ATG5 is knocked down. These results suggested that Beclin 1 and/or UVRAG may regulate centrosome stability independently of autophagy, prevent abnormal centrosome amplification, and maintain genomic stability and normal mitosis 41.

In addition, the E3 ubiquitin ligase parkin can interact with anaphase promoter complex/ cyclosome (APC/C) co-activators Cdc20 and CDH1, and further through the Parkin-Cdc20/Cdh1 complex and the APC/C complex co-regulate mitosis. The deletion of Parkin leads to abnormal levels of mitotic regulators, which may lead to chromosomal instability, the disruption of genomic homeostasis, chromosomal dislocation, and events such as hysteresis, aneuploidy, and cytokinesis defects. These could be closely related to tumorigenesis 99 (Figure 3).

### Non-autophagic function of autophagy-related proteins involved in the cell cycle regulation

Atg7 combines with the tumor suppressor p53 to regulate the transcription of cell cycle inhibitor p21CDKN1A, and p21CDKN1A expression cannot be properly induced in Atg7-deficient cells. The binding of Atg7 and p53 is increased under metabolic stress and starvation. Under starvation, Atg7-deficient cells inhibit p53-mediated cell cycle arrest, whereas under sustained metabolic stress, p53-mediated cell death is increased in Atg7-deficient cells. Therefore, when Atg7 is absent, p53-dependent cell cycle arrest and cell death become more sensitive. The cell cycle defect is independent of the E1-like enzymatic activity of ATG7 and also independent of regulation of autophagy 73. Furthermore, in human bladder cancer (BC), ATG7 knockdown induces cell cycle stagnation in the G2/M phase by promoting P27 expression, which can inhibit tumor development. One possible mechanism is that inhibition of ATG7 expression can stabilize ETS2 mRNA, thus reducing the transcription of mir-196b and the expression of mir-196b. miR-196b can bind to and degrade the 3'UTR of FOXO1 mRNA. Reduced miR-196b expression levels stabilize FOXO1 mRNA and ultimately promote p27 transcription and weaken BC tumorigenic growth 74.

CDC2 regulates the transition from G2 phase to mitosis, and dephosphorylation of CDC2 at Tyr15 is a key step in CDC2 activation 100. Parkin brings about increasing expression of Myt1, followed by phosphorylation of CDC2 at Tyr15, which induces G2/M cell cycle arrest 101. Parkin can not only regulate G2/M cell cycle arrest, but is also the main regulator of G1/S arrest. Knockdown or inactivation of parkin results in massive accumulation of cyclin D1 and cyclin E1, accelerating cell cycle progression 102. Interestingly, parkin mRNA and protein expression are significantly decreased in breast cancer, but no significant changes in cyclin D1 and cyclin E levels are observed, while cyclin-dependent kinase 6 (CDK6) expression level is significantly increased. This suggests an inhibitory role of parkin in the development of breast cancer, and the mechanism may involve a novel association between parkin and CDK6 103. Furthermore, the AKT pathway accelerates G1/S cell cycle progression by increasing cyclin D1 levels, and AKT activation requires T308 phosphorylation of PDK1 and S473 phosphorylation of the TORC2 complex 104. Regulation of VEGF-VEGFR2 on cell cycle progression and cell viability also relate to activation of the PI3K/AKT pathway 105. The study found that parkin could act on TORC2 and significantly affect the expression of VEGFR-2, which suggested that the mechanism of parkin-mediated cell proliferation inhibition could involve the VEGFR2/AKT/cyclin D1 pathway 106.

mTOR also regulates the cell cycle through non-autophagy pathways in response to intracellular and extracellular signals. The regulation of G1 phase progression by mTOR also depends on the activity of the 4E-BP1 and S6K1 pathways, which can regulate the transcription of cyclin D and cyclin E 107. In addition, mTORC1 blocks the nuclear function of p27 KIP1 as a CDK inhibitor by affecting its localization 108. Some studies have found that the phosphorylation of 4E-BP1 and S6K1 still exists during G2/M, and the mTOR pathway also seems to be the guardian of genome stability by regulating the cell cycle during G2/M 109, 110.

As a focal adhesion kinase (FAK) protein inhibitor, FIP200 inhibits FAK kinase activity by binding to FAK, thereby inhibiting its biological function 111. Studies have found that FIP200 can inhibit the G1/S phase progression, cell proliferation, and clone formation of human breast cancer cells. The mechanism is to enhance the activity of the p21 promoter by stabilizing the half-life of upstream p53 and reducing the level of cyclin D1 in breast cancer cells 112.

In addition, it was also reported that the predicted Atg10 homolog (SpAtg10) of the schizosaccharides was essential to maintaining the normal cell cycle process, but it did not combine with Atg12 through autophagic Ubl conjugation pathways 113 (Figure 4) (Table 2).

## Autophagy-related proteins affect human disease by regulating genome stability

### Autophagy-related proteins affect tumor development by regulating genome stability

Autophagy inhibits cell carcinogenesis by maintaining genomic stability in normal cells. However, once the tumor environment is established, autophagy is conducive to the survival and development of cancer cells in the tumor microenvironment 114, 115. During the stage of tumorigenesis, cancer cells lose cell cycle control and exhibit immortal proliferation. Arresting the cell cycle, especially at the G2/M checkpoint, may be an effective method to inhibit tumor progression. The study found that Beclin 1 mutant mice had a relatively high incidence of cancer, including breast tumors, lymphoma, and hepatocellular carcinoma 59, 116. Based on these findings, it can be inferred that that Beclin-1 can block G2/M phase to delay cell cycle progression and induce autophagy and Beclin 1 may be a tumor suppressor factor. The same results have been demonstrated in human cancers, where Beclin 1 expression was reduced in a number of human cancers, including glioblastoma, ovarian cancer, and esophageal cancer 117-119. However, it has been reported that Beclin1 expression is increased in colorectal cancer cells and gastric cancer cells compared with normal cells 120. No biological macromolecule is absolutely good or bad, and these results may suggest that Beclin1 plays distinct roles in different tissues.

UVB and UVA radiation can damage DNA, which is an important cause of skin cancer 121. The tumor suppressor p53 can mediate the apoptosis and clearance of radiation-injured skin cells, but excessive clearance of skin cells can damage the skin barrier function. AKT/mTOR can negatively regulate cell apoptosis, inhibit autophagy and prevent cell cycle arrest, and cells prevent excessive apoptosis of skin cells through the AKT/mTOR anti-apoptotic signaling pathway 122, 123. However, the overactivation of AKT/mTOR will promote the development of epidermal tumors. Therefore, the inhibition of mTOR complex and AKT may be a promising strategy for preventing photocarcinogenesis 124.

Myelodysplastic syndrome (MDS) can transform into acute myeloid leukemia (AML) and is caused by genomic instability and somatic mutations within hematopoietic stem cells (HSPC). Because MicroRNA-146a (miR-146a) is located adjacent to the distal deletion region and the deletion of miR-146a is an initiating event in del (5q) myeloid malignancies (which is dependent on the nuclear factor kappa B (NF-κB) control system), it is related to the pathogenesis of human MDS 125. P62 can recruit TRAF6 and initiate NF-κB signaling. Knockdown of p62 or disruption of p62-TRAF6 binding can lead to cell cycle arrest and apoptosis in MDS/AML cell lines and clinical samples 126. Importantly, in the study, the lack of p62 had little effect on the lifespan and function of normal HSPCs. This suggested that disturbing p62/TRAF6 binding may hold promise for treating miR-146a-deficient leukemia 127.

### Autophagy-related proteins affect cellular senescence by regulating genome stability

Mitotic slippage refers to the cellular process in which mitosis is forced to stall and slip into interphase using antimitotic drugs, when chromosomes do not segregate properly and the cytoplasm does not divide perfectly. Sliding cells may continue to proliferate in a genomically unstable form, or they may arrest in the next post-interphase slip and eventually develop into cellular senescence, with senescence-associated secretory phenotypes (SASP) 128. SASP factors cause paracrine and promote tumor formation. Following cell slippage, autophagy will be induced by ER stress and the AMPK/mTOR/ULK1 axis. Both pharmacological inhibition of autophagy and silencing of ATG5 can prevent the generation of SASP and reduce the paracrine tumorigenicity caused by SASP. Then, cells enter the S phase while inducing DNA damage and replication stress, reducing cell viability, bypassing cellular senescence, accelerating cell death, and preventing tumorigenesis 129.

Furthermore, following DNA damage, cascades signaling of genomic instability are integrated into PGC-1β-dependent mitochondrial biogenesis, which is conducive to ROS-mediated DDR and cell cycle arrest as well as cellular senescence 130. Additionally, there is evidence that reducing mitochondrial content* in vivo* through mTORC 1 inhibition or PGC-1β deletion could delay aging in mouse livers.

## Conclusions and future perspective

The non-autophagic functions of autophagy proteins are receiving increasing attention, among which many functions related to the maintenance of genome stability in the nucleus of autophagy proteins are essential for cell survival. These functions involve DNA damage repair, protection of chromosome stability, and cell cycle regulation. We summarized the roles of autophagy proteins in maintaining genome stability in the nucleus through both autophagy-dependent and autophagy-independent pathway. Some autophagy proteins can act through both autophagy-dependent and autophagy-independent pathways, while others can only act through a certain pathway. These autophagy proteins are closely related to tumorigenesis and development of various tumors and the process of cell senescence, which could open door to new ideas for the prevention and treatment of diseases. This enriches the background of these autophagic proteins, indicating a non-autophagic function in the nucleus. Research on the non-autophagy function of autophagy proteins is currently a hot issue among scientists. Therefore, fully revealing their functions, especially their non-autophagic functions, could lead to new theories and in-depth understanding of the biological significance of autophagy-related proteins.

## Figures and Tables

**Table 1:** Autophagy-related proteins involved in genome stability through autophagy-dependent pathway

Autophagy-related proteins	Target molecule	Functions	References
p62	RNF168	reduces the recruitment of DNA repair protein	45
	FLNA and RAD51	inhibits HR-directed DSB repair	49
ULK1/LC3	P53, γ -H2AX, Rad51, PARP-1	promote DSB repair	36
Beclin1	UVRAG	is involved in DDR and protect chromosome stability	40, 41
	PLK1 and CDC25C	is involved in IR-induced G2/M arrest	68
ULK1/ATG13	CDK1-CCNB/cyclin B	are involved in cell cycle progression and cell proliferation	69, 70
ATG1/ATG18		protect chromosome stability	56
ATG7		accelerates the progression of cells entering S phase	72
		deficiency of ATG7 blocks G1/S, G0/G1 phase	75, 76
ATG10		promotes cell proliferation	77
ATG4	LKB1-AMPK	inhibits the progression of cells entering the G1/S transition	78
mTORC1	cyclin D1	mTORC1 inhibition activates autophagy and induces G1 cell cycle arrest	79, 80
PINK1/parkin	TBK1	inhibits the progression of cells entering the G2/M transition	81
FIP200		is involved in DNA repair	38

**Table 2:** Autophagy-related proteins involved in genome stability through non-autophagic pathway

Autophagy-related proteins	Target molecule	Functions	References
mTORC1	S6K/FANCD2, 4E-BP1	promotes DNA repair and replication fork survival	81,82
	4E-BP /S6K1/cyclin D and cyclin E	regulates G1 phase progression	107
	S6K1	stabilizes the genome in G2/M phase	109
ATG5	Mis18α	microsatellite instability and DNA mismatch repair deficiency	93
p62	DLK1/NADPH oxidase/ROS	induces genomic instability	95
ULK1	PARP-1	is as a DNA damage factor	96
Beclin1	DNA topoisomerase IIβ/53bP1	is involved in DNA repair	97
	Zwint-1	Protects chromosomestability	98
ATG7	p53	Regulates cell cycle during metabolic stress;	73
	ETS2/miRNA196b/FOXO1/p27 Axis	Knockdown of ATG7 induces cell cycle arrest in G2/M phase.	74
parkin	Cdc20/Cdh1/APC/C	Protects chromosomestability	99
	Myt1/CDC2	Induces cell cycle arrest in G2/M phase;	101
	cyclin D1 and cyclin E1	Knockdown or inactivation of PARK2 accelerates cell cycle progression;	102
	VEGFR2/Akt/cyclin D1	Promoted G1 phase cell-cycle arrest and mitigated the proliferation.	106
FIP200	p21/cyclin D1	Inhibits G1-S phase progression and cell proliferation	112
SpATG10		Is essential for normal cell cycle progression in S. pombe.	113

## Abbreviations

AML: acute myeloid leukemia
APC/C: anaphase promoter complex/ cyclosome
BC: bladder cancer
CIN: Chromosome instability
CCD: coiled-coil domain
CIMP: CpG island methylation phenotype
CDK6: cyclin-dependent kinase 6
CDKs: cyclin-dependent kinases
DDR: DNA damage response
DSB: DNA double-strand break
FLNA: filamin A
FAK: focal adhesion kinase
GC: gastric cancer
HSPC: hematopoietic stem cells
HR: homologous recombination
IR: ionizing radiation
LB: LIM-binding
METTL14: methyltransferase-like 14
miR-146a: microRNA-146a
MMR-D: mismatch repair deficiency
MLH1: mutL homolog 1
MDS: Myelodysplastic syndrome
m6A: N6-methyladenosine
NSC: neural stem cells
NHEJ: non-homologous end joining
NF-κB: nuclear factor kappa B
53bP1: p53-binding protein 1
PAS: pre-autophagy structures
PKB/AKT: Protein kinase B
ROS: reactive oxygen species
RZZ: Rod-ZW10-Zwilch
SASP: senescence-associated secretory phenotypes
Ulk1: Unc-51-like kinase 1
